# Maintenance Therapy of Psychosis Spectrum Disorders in a Real-World Setting: Antipsychotics Prescription Patterns and Long-Term Benzodiazepine Use

**DOI:** 10.3389/fpsyt.2022.796719

**Published:** 2022-04-07

**Authors:** Nadja P. Maric, Sanja Andric Petrovic, Manuela Russo, Stefan Jerotic, Ivan Ristic, Bojana Savić, Tamara Pemovska, Milos Milutinovic, Emina Ribic, Silvana Markovska-Simoska, Alma Dzubur Kulenovic, Nikolina Jovanovic

**Affiliations:** ^1^Faculty of Medicine, University of Belgrade and Institute of Mental Health, Belgrade, Serbia; ^2^Unit for Social and Community Psychiatry, WHO Collaborating Centre for Mental Health Services Development, Queen Mary University of London, London, United Kingdom; ^3^Faculty of Medicine, University of Belgrade and Clinic for Psychiatry, University Clinical Centre of Serbia, Belgrade, Serbia; ^4^University Clinic of Psychiatry, Ss. Cyril and Methodius University in Skopje, Skopje, North Macedonia; ^5^Department of Psychiatry, Clinical Centre of the University of Sarajevo, Sarajevo, Bosnia and Herzegovina; ^6^Macedonian Academy of Sciences and Arts, Skopje, North Macedonia

**Keywords:** psychosis spectrum disorders, guidelines, antipsychotic, benzodiazepine, sex-differences

## Abstract

**Background:**

Maintenance therapy of patients with primary psychosis spectrum disorders (PSD) in the Western Balkans has received limited interest so far. The present study aimed to investigate long-term prescription patterns among outpatients with PSD.

**Methods:**

Information about prescription of antipsychotics (AP), benzodiazepines (BZD) and other psychotropic medication over a 6-month period was collected from outpatients (*n* = 134; ICD-10 diagnosis F20-29) recruited by a larger multi-site study, to find mean daily number of psychotropic drugs, AP prescription patterns (including AP daily dose, route of administration, monotherapy *vs*. polypharmacy) and BZD utilization (long-term add-on BZD therapy). Additionally, sex-differences in the variables were explored.

**Results:**

Clinically stable outpatients (age 41.7 ± 11.0; male 62.7%; duration of untreated illness 12.7 ± 8.7 years; mean number of lifetime hospitalizations 2.6 ± 0.7) were prescribed 2.8 ± 1.1 psychotropic medications daily. The mean 6-month AP dose was 14.2 ± 7.8 mg olanzapine equivalents. Long-acting injectable AP was prescribed to 25.2% of the patients. Long-term AP monotherapy was found in 52.7% patients and most of them were prescribed second generation AP (65.2%). Long-term AP polypharmacy (42.7%) was more common in males (*p* = 0.015). The most frequent co-prescription patterns were first generation AP plus clozapine. The highest rate of long-term AP co-prescription was found for BZD (in 42.7% cases, average 6-months daily dose of 2.8 ± 2.7 mg lorazepam equivalents) and anticholinergics (33.6%).

**Conclusion:**

Existing appropriately designed interventions aiming to safely switch the inappropriate therapeutic regimens, i.e. very high prevalence of long-term AP polypharmacy and non-rational BZD co-prescription, should be implemented in the region of Western Balkans.

## Introduction

Antipsychotic (AP) maintenance treatment (i.e., continuous treatment with the lowest effective dose of oral or long-acting AP medication) is recommended to prevent relapses in persons with psychosis spectrum disorders (PSD) according to contemporary drug prescribing treatment guidelines (1, 2). Guidelines usually recommend that AP monotherapy (APM) should be favored. AP polypharmacy (APP) is considered appropriate for short periods of cross-titration (AP switching), cases of higher clinical complexity and treatment resistance, or to combine different pharmacological effects (3, 4). However, real-world prescribing patterns often differ from guidelines' suggestions.

The maintenance therapy of outpatients with PSD may include simultaneous prescription of two or more APs or co-prescription of AP with other psychotropic drugs. Such prescribing practices may vary across countries due to different factors, such as patient-level issues (mental health literacy, stigmatization), provider prescribing decisions, factors beyond medication effectiveness (order of introduction, ease of use, prescribers' idiosyncratic beliefs, marketing efforts), local culture and many other factors (5). Nevertheless, psychiatrists who treat patients with psychotropic polypharmacy should be aware of potential drug interactions. Studies examining the safety of these combination treatments practically do not exist.

Even though prescription guidelines do not differentiate between male and female patients, there are recommendations that optimal maintenance regimes of AP might not be the same for both sexes due to different pharmacokinetics and pharmacodynamics of AP drugs (6, 7). Male sex could be associated with greater use of APP, mood stabilizers (MS) and anticholinergic medication (ACM), as it was shown in Asia by Xiang et al. (8), while females could be in a higher risk of irrational benzodiazepine use (9). This could vary (10, 11) and sex-differences in psychotropic drug prescription patterns have to be studied to address many potential sex- specific considerations in psychotropic drug utilization (12).

Mental health-care practice in Central and Eastern European countries remains ‘a blind spot on the global mental health map'. According to Winkler et al. (13), the decision making on resource allocation is not transparent, epidemiological surveys are lacking and stigma seems to be higher than in other European countries. Research into long-term pharmacotherapy patterns of patients with PSD is sparse. Given that the most commonly used methods for identifying psychotropic polypharmacy prescriptions could fail to differentiate between the appropriate prescriptions of multiple psychotropic agents on a short-term temporary basis and long-term treatment regimens (14), the present study was designed to overcome this limitation by collecting detailed information on six-months psychotropic prescribing patterns.

The main goal of the present study was to elucidate trends of long-term APP, psychotropic polypharmacy and long-term BZD use in the maintenance therapy of outpatients with PSD in the Western Balkans, where the citizens have universal free access to healthcare and most of the first and second generation antipsychotic drugs, clozapine and BDZs are fully reimbursed. The secondary goal was to explore potential sex-differences in the psychotropic medication prescribing practices.

## Materials and Methods

Present cross-sectional study used retrospective medical chart review and analyzed prescription patterns for PSD outpatients. The study sample was derived from the Western Balkans' countries involved in the multicentric IMPULSE study (15) exploring the implementation of the psychosocial intervention DIALOG+ for patients with PSD in low- and middle-income countries in Southeast Europe (Grant agreement no.779334) (15). The inclusion criteria were the same as those for the IMPULSE study (16) participants (*n* = 425): outpatients with primary diagnosis of PSD (ICD-10 codes F20-29), age 18–65 years, history of at least one psychiatric hospital admission during lifetime, capacity and will to provide informed consent and available medical charts at the time of assessment. Exclusion criteria were: diagnosis of organic brain disorders, severe cognitive deficits and inability to provide informed consent, incomplete medical documentation or any other data relevant for the present study. Additionally, for the purposes of the present study we collected information concerning prescription patterns in PSD outpatients over the observed 6-month period. The final sample consisted of 134 outpatients from six study sites in Bosnia and Herzegovina, Republic of North Macedonia and Serbia (two sites per country). The remaining participants from the IMPULSE study (*n* = 291) were not included in this particular analyses due to incomplete medical charts. The study was conducted in accordance with the Declaration of Helsinki and its design was approved by the Ethics Committees of the participating countries. All participants provided informed consent before the initiation of the study.

### Clinical Assessment

Medical chart review was used to obtain age at diagnosis, duration of illness and number of psychiatric hospitalizations. Duration of illness was counted as the year since psychotic disorder was first diagnosed, whereby the date of diagnosis was used as the reference point. The number of hospitalizations included only inpatient admissions (day hospital admissions were omitted). Symptom severity at the time of the evaluation was measured using the 24-item Brief Psychiatric Rating Scale (BPRS) (17) and the 13-item interview-based questionnaire Clinical Assessment Interview for Negative Symptoms (CAINS) (18). For both scales lower scores reflected less severe psychopathology.

### Medication Related Variables

Retrospective medical chart review was used to list all psychotropic medications prescribed over the 6-months period preceding the clinical evaluation – generic and trade names of psychotropic drugs (antipsychotics (AP), antidepressants (AD), mood-stabilizers (MS), benzodiazepines (BZD), anticholinergic medication (ACM), drugs used to treat addictive disorders) and non-psychotropic concomitant medication, psychotropic drug daily doses (DD), use on a regular basis or discontinuously -as needed. Several variables related to psychotropic prescription patterns were calculated for the purposes of the present analyses, with particular focus on AP and BZD prescriptions.

### General Psychotropic Medication Prescription Patterns

- Mean daily number of psychotropic medications – an average number of psychotropic medications prescribed on a daily basis over the observed 6-month period.

### AP Prescription Patterns

- Groups of AP: First-generation agents – FGA (chlorpromazine, promazine, fluphenazine, haloperidol, levomepromazine, sulpiride, zuclopenthixol); Second-generation agents – SGA (risperidone, olanzapine, quetiapine, paliperidone): Third generation agents – TGA (aripiprazole), and Clozapine (separate class as clozapine is the only third line therapy agent with a unique mechanism of action).
- Predominant AP group – the AP group prescribed longer and/or in higher daily doses during the observed 6-months period, in comparison to the other prescribed AP groups.
- The most commonly prescribed AP – not taking into account the whole prescription pattern (i.e., single use or multiple concurrent AP use).
- Mean daily AP dose – the average six-month daily dose (DD) transformed into the olanzapine (OLA) equivalents (19, 20). In cases with more than one AP, their OLA equivalents were summed to obtain the total average AP DD. According to the World Federation of Societies of Biological Psychiatry (WFSBP) Guidelines (9), AP DD above 600 mg chlorpromazine (CPZ) equivalents (19.8 mg OLA equivalents) were considered high maintenance doses. More recently, the Canadian Psychiatric Association – CPA (21) lowered the upper limit of the recommended CPZ equivalent dose from 600 mg/day to 400 mg/day (13.2 mg OLA equivalents). Both WFSBP and CPA thresholds were used in the further considerations.
- Route of AP administration – the binary categorical variables (yes/no) described AP formulation delivered over the observed six-month period: (1) Long-acting injectable (LAI) / depot-only – AP only prescribed through depot / LAI formulation; (2) Oral-only – only oral AP formulation was used; (3) LAI / depot-predominantly – LAI / depot prescribed in higher average dose and/or for a longer time period over the observed 6 months in comparison to the oral AP, and 4) Oral-predominantly – oral AP formulation was in higher average dose and/or for a longer time period in comparison to the LAI / depot formulation over the 6-months observation.
- Rate of monotherapy *vs*. polypharmacy – the binary categorical variables (yes/no) described AP drug prescription patterns: (1) long-term AP monotherapy (APM) – AP monotherapy continuously prescribed for at least 6 months, (2) long-term AP polypharmacy (APP) – concurrent continuous use of more than one AP for at least 6 months, (3) uncategorized AP prescription pattern – combined APM/APP prescription during the observational period (the defined criteria for APM/APP were not fulfilled). Long-term add-on therapy – Combinations of AP with other psychotropic drugs (AD/MS/BZD/ACM) continuously prescribed for at least 6 months.

### BZD Prescription Patterns

- Long-term BZD prescription – the rate of patients prescribed BZD continuously over the observed 6 months period (BZD long-term group) was calculated and this group has been compared with other patients (BZD-other). Later group involved patients with no use, or any BZD use lasting <6 months.
- Mean daily BZD dose – during the observed 6-months period average daily BZD doses were calculated and transformed into lorazepame quivalents (22). Lorazepam 1 mg equivalent doses: diazepam 5 mg, bromazepam 3 mg, clonazepam 0.5 mg, alprazolam 0.25 mg, midazolam 7.5 mg, zolpidem 5 mg, nitrazepam 5 mg (midazolam, zolpidem and nitrazepam were considered as hypnotics, with others considered as anxiolytics). The equivalent doses have been derived from two resources (23, 24), as per previous articles (25). In cases where more than one BZD was prescribed, their lorazepam equivalent doses were summed to obtain the total BZD DD. According to the ATC/DDD system, the mean daily dose >2.5 mg of lorazepam equivalents (DDD) was considered high.
- Long-acting vs. short-acting BZD: according to the Longo and Johnson (26), clonazepam and diazepam were considered long-acting, and all other short-acting drugs.

### Statistical Analyses

All statistical analyses were performed by the SPSS version 20.0 statistical software. Descriptive statistics (sociodemographic and clinical measures) were presented as absolute and relative numbers, means, standard deviations and medians. Before between-group analyses, normality was tested using the Shapiro-Wilk test. The only variables that were normally distributed were age at the time of the assessment and AP DD. The remaining continuous variables that did not have a normal distribution were: number of previous psychiatric hospitalizations, number of psychotropic drugs prescribed on a daily basis, BZD DD, age at the time of diagnosis, illness duration. The between-group differences were tested using the appropriate parametric or non-parametric tests (Chi-square test, Mann-Whitney test, Student *t*-test for independent samples). Effect sizes were interpreted as follows: Mann– Whitney r– 0.1 small, 0.3 medium, 0.5 large; Cramer's V−0.06 small, 0.17 medium, 0.29 large; Cohen's D−0.2 small, 0.5 medium, 0.8 large. All *p* < 0.05 were considered significant.

## Results

### Description of the Sample

The main socio-demographic and clinical characteristics of the sample are presented in Table 1.

**Table 1:** Sociodemographic and clinical characteristics of the sample.

**Age (years), mean ± SD**	**41.7 ± 11.0**
**Sex (male), n (%)**	**84 (62.7)**
**Education, n (%)**	
**Elementary school**	**18 (13.4)**
**High school**	**90 (67.2)**
**University/college/postgraduate**	**26 (19.4)**
**Employment, n (%)**	
**In paid employment**	**28 (20.9)**
**Retired**	**31 (23.1)**
**Other (student, unemployed)**	**75 (56.0)**
**Marital status, n (%)**	
**Married/partnership**	**26 (19.4)**
**Other (single, widowed, divorced)**	**108 (80.6)**
**Diagnosis (ICD-10), n (%)**	
**Schizophrenia – F20**	**78 (58.2)**
**Other (F21, F22, F23, F25, F29)**	**56 (41.8)**
**Age at diagnosis (years), mean ± SD**	**29.3 ± 8.2**
**Duration of illness (years), mean ± SD**	**12.7 ± 8.7**
**Number of hospitalizations, mean ± SD**	**2.6 ± 0.7**
**BPRS^a^ total average score, mean ± SD**	**1.6 ± 0.5**
**CAINS^b^ score, mean ± SD**	**1.5 ± 0.8**

a *BPRS, Brief Psychiatric Rating Scale;*

b *CAINS, Clinical Assessment Interview for Negative Symptoms*.

### Prescription Patterns

The mean total number of psychotropic medications prescribed to the PSD outpatients over the 6 months was 2.8 ± 1.1, whereby 13.4% patients were prescribed one psychotropic drug. Psychotropic polypharmacy was found in all other patients: 22.4% were prescribed with two psychotropic drugs, 32.8% with three, 19.4% with four and 7.5% with five psychotropic drugs. The most common long-term AP add-on therapy were BZDs (42.7%), followed by anticholinergics (33.6%). For detailed description of the psychotropic drug prescription patterns among the sample (see Table 2).

**Table 2:** Psychotropic drug prescription patterns among the sample.

**Daily number of psychotropic medications over the**	**2.8 ± 1.1 (3)**
**6 months, mean ± SD (MED)**	
**AP^a^ prescription patterns, n (%)**	
**Long term APM^b^**	**69 (52.7)**
**Long term APP^c^**	**56 (42.7)**
**Uncategorized AP^a^ prescription pattern**	**6 (4.6)**
**Predominant AP^a^ group, n (%)**	
**First generation AP^a^**	**30 (22.9)**
**Second generation AP^a^**	**62 (47.3)**
**Third generationAP^a^**	**7 (5.3)**
**Clozapine**	**29 (22.1)**
**Mixed (equal doses)**	**3 (2.3)**
**Route of administration, n (%)**	
**LAI^d^/depo-only**	**9 (6.9)**
**Oral-only**	**98 (74.8)**
**LAI^d^/depo predominantly**	**6 (4.6)**
**Oral predominantly**	**18 (13.7)**
**Long term AP^a^ add-on therapy, n (%)**	
**Antidepressant**	**24 (18.3)**
**Mood stabilizer**	**34 (26.0)**
**Anticholinergic**	**44 (33.6)**
**Benzodiazepine**	**56 (42.7)**
**Mean AP^a^ daily dose (OLA^e^ equivalents, mg), mean ± SD**	**14.2 ± 7.8**
**High AP^a^ maintenance dose, n (%)**	
**WFSBP^f^ criteria (above 19.8 mg OLA^e^ equivalents)**	**34 (26.0)**
**CPA^g^ criteria (above 13.2 mg OLA^e^ equivalents)**	**68 (51.9)**

a *AP, antipsychotic;*

b *APM, antipsychotic monotherapy;*

c *APP, antipsychotic polypharmacy;*

d *LAI, long-acting injectable;*

e *OLA, olanzapine;*

f *WFSBP, World Federation of Societies of Biological Psychiatry;*

g *CPA, Canadian Psychiatric Association*.

#### Antipsychotic Prescription

In total, 131 patients were prescribed AP during the whole observed period (97.8%), with an average daily dose of 14.2 ± 7.8 mg OLA equivalents. One patient diagnosed with brief psychotic disorder (F23) was not prescribed any psychotropic agent and two patients (schizophrenia – F20 and schizoaffective disorder – F25) were treated with a combination of AD, MS and BZD.

The most commonly prescribed AP for any prescription pattern (including both APM/APP and long-term/short-term prescription patterns) in the whole sample was clozapine (35.1%), followed by haloperidol (26.7%), risperidone (26.0%), olanzapine (21.4%), aripiprazole (10.7%), paliperidone (9.9%), fluphenazine (7.6%), chlorpromazine (5.3%), promazine (5.3%), levomepromazine (3.8%), quetiapine (3.1%), zuclopenthixol (0.8%) and sulpiride (0.8%).

In the subsample of 46 participants prescribed clozapine it was predominant AP in 29/46 patients (and the only AP prescribed in 13 of them), and it was add-on AP to the other predominant AP in 17/46 patients. Patients prescribed clozapine had more previous psychiatric hospitalizations (*p* = 0.000, *r* = 0.43), more psychotropic drugs daily (*p* = 0.009, *r* = 0.23), they were more frequently treated with APP (*p* = 0.000, Cramer's V = 0.407), had more add-on BZD therapy (*p* = 0.021, Cramer's V = 0.216) and higher BZD DD (*p* = 0.017, *r* = 0.21) in comparison to the non-clozapine group. In terms of route of AP administration over the observed 6-months-period, 25.2% of the patients were prescribed LAI/depo AP (33/131), whereby 15/33 were exclusively/predominantly treated with LAI/depot formulation.

Long-term APM was found in 52.7% and most of them were prescribed SGA (45/69 patients). The average daily AP dose in the APM group was 10.8 ± 5.9 mg OLA equivalents. Long-term APP was observed in 42.7% patients with the average daily AP dose of 18.0 ± 8.3 mg OLA equivalents, and the most frequent combination was FGA plus Clozapine co- prescription (24/56). Out of the remaining nine patients (6.7% of the sample) – three patients were not prescribed AP and six patients had the uncategorized AP prescription pattern (i.e., combined APM/APP prescription not meeting the defined criteria for APM/APP).

Significantly less long-term APP was observed in women (*p* = 0.015, Cramer's V = 0.234), in patients with fewer hospitalizations (*p* = 0.001, *r* = 0.30), in those without continuous long-term add-on ACM therapy (*p* = 0.018, Cramer's V = 0.228) and in patients prescribed lower average BZD DD (*p* = 0.026, *r* = 0.192). In patients with long-term APP significantly higher AP DD were prescribed (*p* = 0.000, Cohen's D = 0.998) and they were more frequently prescribed above the recommended average AP DD (WFSBP criteria >19.8 OLA equivalents – *p* = 0.000, Cramer's V = 0.377; CPA criteria >13.2 OLA equivalents – *p* = 0.036, Cramer's V = 0.204) in comparison to the APM group. In APP patients, diagnosis of schizophrenia (F20) was more common than other PSD (in comparison to the APM group) at the level of trend (*p* = 0.056, Cramer's V = 0.187). The trend of more common prescription of LAI/depo medication was also observed in the APP group (*p* = 0.088, Cramer's V = 0.172).

For more information about the between-group differences (i.e., APM *vs*. APP) (see Table 3).

**Table 3:** Antipsychotic and benzodiazepine prescription patterns and their relation to sociodemographic and clinical parameters.

	**Antipsychotic**			**Benzodiazepine**		
	**APM^a^ (*n =* 69)**	**APP^b^ (*n =* 56)**	***p***	**BZD^f^- other (*n =* 78)**	**BZD^f^ long-term use (*n =* 56)**	***p***
Age (years), mean ± SD	41.8 ± 11.5	41.2 ± 10.8	0.771	38.6 ± 10.9	46.1 ± 9.7	0.001^**^
Sex (male), n (%)	36 (52.2)	42 (75.0)	0.015^*^	52 (66.7)	32 (57.1)	0.346
Diagnosis (ICD-10), n (%) Schizophrenia – F20	34 (49.3)	38 (67.9)	0.056	41/78	37/56	0.166
Other (F21, F22, F23, F25, F29)	35 (50.7)	18 (32.1)		37/78	19/56	
Age at diagnosis (years), mean ± SD	30.2 ± 9.0	27.7 ± 7.0	0.091	27.8 ± 7.6	31.4 ± 8.7	0.012^*^
Duration of illness (years), mean ± SD	11.9 ± 8.9	13.9 ± 8.5	0.127	11.3 ± 8.8	14.7 ± 8.4	0.009^**^
Number of hospitalizations, mean ± SD	1.1 ± 11.1	4.2 ± 3.7	0.001^**^	2.7 ± 3.4	4.1 ± 3.0	0.001^**^
LAI^c^/Depot AP^d^, n (%)	11 (15.9)	17 (30.4)	0.088	N/A	N/A	
Total number of psychotropic medication	2.3 ± 1.0	3.6 ± 0.9	0.001^**^	2.2 ± 0.9	3.6 ± 0.9	0.001^**^
Mean AP^d^ daily dose (OLA^e^ equivalents, mg), mean ± SD	10.8 ± 5.9	18.0 ± 8.3	0.001^**^	12.6 ± 7.01	15.8 ± 8.6	0.021^*^
Mean BZD^f^ daily dose (lorazepam equivalents, mg), mean ± SD	2.3 ± 1.9	3.5 ± 3.3	0.026^*^	1.1 ± 0.8	3.2 ± 2.9	0.001^**^

a *APM, antipsychotic monotherapy;*

b *APP, antipsychotic polypharmacy;*

c *LAI, long-acting injectable;*

d *AP, antipsychotic;*

e *OLA, olanzapine;*

f *BZD, benzodiazepine.*

* *p < 0.05;*

** *p < 0.01; statistically significant values are bolded*.

#### Prescription of Benzodiazepines

The most commonly prescribed BZD was diazepam (22.4%), followed by lorazepam (11.9%), clonazepam (6.0%), bromazepam (5.2%), alprazolam (3.0%), zolpidem (2.2%), midazolam (1.5%) and nitrazepam (0.7%). More than a half of the patients with continuous long-term BZD use were prescribed long half-life agents (33/56 patients). For more information regarding BZD prescription patterns (see Table 3).

Patients with BZD prescribed continuously over the observed 6 months (56/134, 41.8% of the sample) were significantly older both at the time of diagnosis (*p* = 0.012, *r* = 0.216) and at the time of the assessment (*p* = 0.000, Cohen's D = 0.731), had longer illness duration (*p* = 0.009, *r* = 0.225) and more lifetime psychiatric hospitalizations (*p* = 0.000, *r* = 0.310) in comparison to other group. Long-term BZD use was associated with more psychotropic polypharmacy (*p* = 0.000, *r* = 0.62), with higher average AP DD (*p* = 0.021, Cohen's D 0.406), higher average BZD DD (*p* = 0.000, *r* = 0.318) and more frequent additional “as needed” BZD prescription (*p* = 0.005, Cramer's V = 0.265).

### Sex-Differences

Our PSD sample consisted of more males (*p* = 0.003), who were younger at the time of the first diagnosis in comparison to females (28.1 ± 7.7 *vs*. 31.3 ± 8.8 years, *p* = 0.037, *r* = 0.18), however at the time of assessment the two groups did not differ in terms of age, illness duration, diagnostic categories, number of hospitalizations. Except showing that females had significantly less APP (see above), no other sex-differences were detected in the prescription patterns – mean number of prescribed psychotropic medications (*p* = 0.338), LAI/depot AP formulation usage (*p* = 0.114), average AP DD (*p* = 0.850), rate of BZD-long term cases (*p* = 0.346) and average BZD DD (*p* = 0.448).

## Discussion

To the best of our knowledge, this is the first study in the Western Balkan to show long-term psychotropic prescription patterns in clinically stable real-world patients with PSD. Their prescription included on average 2.8 different psychotropic medications, with APP in 42.7% cases – which could be considered a very high rate of APP. The average AP DD found in the present study was moderate for the group as a whole. However, combination of APs was not associated with reduced dose of individual APs. In the long-term APM group, the SGA were the most common choice and this treatment regimen was associated with lower AP DD and less frequent add-on BZD or ACM. APM patients had less lifetime hospitalizations in comparison to APP, suggesting either lower clinical complexity of the primary condition, or a better functioning and lower rate of complications due to rational psychotropic use in line with clinical recommendations. In the systematic review of global and regional trends from 1970s to 2009, Gallego et al. (27) showed median APP rate of 19.6%, but the authors emphasized a wide variation across countries (interquartile range = 12.9–35.0%). Lin (28) discussed these findings and confirmed that APP rates differ between regions – higher rates were found in Asia and Europe in comparison to North America, Australia and Oceania. Interestingly, during the last few decades, in some of these regions the rate of APP increased (NorthAmerica), while in Asia it decreased. No similar changes have been found in Europe, where APP rate has been approximately 23% throughout the observed period. In a general population in Grece, Pappa et al. (29) showed that polypharmacy mainly depended on health needs, followed by education, utilization of health services and age. The rate of the observed APP *vs*. APM in the present study was higher in comparison to data from large-scale scale studies worldwide (30, 31), but in line with the recent finding from Serbia for the 1-month observational period (prevalence of APP was around 45%) (32).

The common practice of APP in schizophrenia and other primary psychoses still lacks double- blinded or high-quality evidence of efficacy in the maintenance phase. One exception is the Scandinavian study (33), where aripiprazole & clozapine combination was shown to decrease the risk of rehospitalization (marker of relapse) – indicating that certain types of polypharmacy (for example, two particular APs with different types of receptor profiles), maybe feasible for the treatment of schizophrenia. Nevertheless, in most of the APP cases such prescription patterns should be used to identify treatment problems and to stimulate search for the alternatives to polypharmacy (34).

According to the case-mix models, APP has been used more often for individuals with chronic disease and more thought disturbance, but it has been only partly a reaction to disease severity (34). Other reasons for APP include combining complementary pharmacologic and side effect profiles, treatment of different symptom domain, treatment of comorbidity, or prescribers' habits/skepticism toward the use of treatment guidelines. Importantly, in the majority of patients and in a various clinical circumstances it is possible to reduce therapy/switch to APM without worsening the outcomes (35). There are proven effective and appropriately designed methods of reducing APP (36, 37) that could be implemented.

As opposed to the finding of higher APP rate in the present sample, the use of LAI/depot AP formulation in approximately 25% participants seems somewhat lower than reported in the literature. Previous study demonstrated that about one-third of patients with schizophrenia were poorly adherent to oral medications, which differs from our findings, but one possible explanation could be that the present study involved more compliant patients (willing to adhere to the IMPULSE protocol), or less schizophrenia patients in comparison to other studies of LAI formulations (38).

The proportion of individuals with psychosis prescribed at least one AP did not differ between the Western Balkans and the UK (97.7 *vs*. 97.5%, respectively) (39). On the contrary, the utilization of clozapine in the present sample was higher in comparison to the U.S. (40) or the UK (41) suggesting low levels of “clozaphobia” (42) in the Western Balkans. Clozapine is not an expensive drug, blood monitoring is fully covered by the national health insurance and dispensing requirements for it are less restrictive in comparison to the western countries, which might have impact on its prescribing and wider usage. The finding that clozapine was associated with more previous psychiatric hospitalizations is expected and indirectly confirmed an appropriate choice of the third-line medication, however the fact that this pharmacologically “rich” molecule was prescribed with many other psychotropic medications and rather in APP than in APM (the difference with high effect size) needs further consideration.

Besides high rates of APP, our analysis of PSD maintenance therapy showed an alarming number of patients with long-term BZD use, which could be associated with serious health risks. In the large-scale study from Denmark, the risk of natural death did not increase in relation to APP prescription patterns compared with APM (43), however the combination of AP with long half-life BZD was associated with an increased risk of natural death. Similarly, when Tiihonen et al. (44) explored the association of mortality and psychotropic polypharmacy in Finland (all-cause mortality during the use of AP, AD and BZD), a substantial increase in mortality- attributable to both suicidal and non-suicidal deaths – was associated with the combination of BZD and AP. Long-term use of BZD has been shown to increase the risk of accidents, suicide attempts, reduction of the work capability, and the costs of hospitalization (45). Therefore, the finding of excessive rate of patients continually co-prescribed with long-term BZD in the present study (42.7%), requires strategies to increase the knowledge about rational BZD use in the region (25).

The rate of patients continually prescribed ACM for at least 6 months in this study (33.6%) could be also considered high. Short-term/when needed use of anticholinergic agents alleviate AP-induced extrapyramidal side-effects, but their long-term use might increase risk of adverse cognitive effects (especially in elderly) (46) and has abuse potential (47). The fact that its use is becoming routine and commonplace (48) calls for targeted intervention, too.

Finally, given that the subjective tolerability of AP drugs could substantially differ between sexes (49), this study showed lower rate of APP in females which was similar to findings from Asia (9). PSD patients of both sexes from the Western Balkans had almost same average AP DD (approximately 14 mg of OLA equivalents), similar proportions of high maintenance AP daily dose and almost equal long-term BZD daily dose. According to Seeman (6), women's psychotic symptoms respond to AP drugs at doses lower than men's which means that many women in our sample might possibly be overdosed, experiencing unnecessary adverse effects. However, lower rates of APP in females could counteract possible higher AP DD effects.

This study has several strengths and limitations. Firstly, it was a convenience sample of the whole spectrum of non-affective psychosis cases (ICD-10 codes F20-29) from six outpatient sites in the region, therefore for concluding about the specific diagnostic subgroup or distinguishing between patterns of combination therapies, or between particular countries, this sample was underpowered. Thus, our findings may not be generalizable to the region as a whole. Also, the licensing and availability of different medicines throughout Europe and internationally limits the transferability of the results. Secondly, our sample included 37.3% females and it is unknown if this completely reflect sex ratio of PSD in the region. Although males outnumber females almost two times in the non-epidemiological studies of the PSD patients (50), this issue should be taken into account for future research. Third, the conversion of psychotropic drug doses into OLA equivalents and its accuracy (in particular depot AP) has been a general problem with prescription pattern surveys (51). However, averaging CPZ seems to be even a less scientific approach which is more prone to bias, because studies reported that many CPZ equivalents reported in publications were not based on evidence, but rather on the clinical experience of the authors (9). Finally, this study analyzed prescription patterns only, which is not always the same as the medication used by patients. Nevertheless, to the best of our knowledge, this is the first study in Western Balkan analyzing 6-months prescription patterns in a carefully monitored group of stabilized PSD patients. Present study overcame the limitations of similar studies of AP that have focused on short-term polypharmacy which could reflect cross-titration and varying clinical conditions. Using a wide range of well-defined variables, this study is providing comprehensive information about the maintenance therapy of PSD in a real world setting.

Present findings reveal distinct prescription patterns concerning APP and long-term BZD use in the Western Balkans, and this information could be used for future analyses of the real- world practices and for the targeted, properly designed interventions for improving life of persons with psychosis spectrum disorders.

## Data Availability Statement

The raw data supporting the conclusions of this article will be made available by the authors, without undue reservation.

## Ethics Statement

The studies involving human participants were reviewed and approved by the Ethics Committees of the Faculty of Medicine University of Belgrade and all other participating countries. The patients/participants provided their written informed consent to participate in this study.

## Author Contributions

All authors contributed to the study conception and design, material preparation, data collection, commented on previous versions of the manuscript, and read and approved the final manuscript.

## Funding

Results of this study are a part of the IMPULSE project. The IMPULSE project has received funding from the European Union's Horizon 2020 research and innovation program under Grant Agreement No. 779334. The funding was received through the Global Alliance for Chronic Diseases prevention and management of mental disorders (SCI-HCO-07-2017) funding call.

## Conflict of Interest

The authors declare that the research was conducted in the absence of any commercial or financial relationships that could be construed as a potential conflict of interest.

## Publisher's Note

All claims expressed in this article are solely those of the authors and do not necessarily represent those of their affiliated organizations, or those of the publisher, the editors and the reviewers. Any product that may be evaluated in this article, or claim that may be made by its manufacturer, is not guaranteed or endorsed by the publisher.
